# Cytotoxicity and Degradation Resistance of Cryo- and Hydrogels Based on Carboxyethylchitosan at Different pH Values

**DOI:** 10.3390/gels10040272

**Published:** 2024-04-17

**Authors:** Elena Blinova, Anastasia Korel, Ekaterina Zemlyakova, Alexander Pestov, Alexander Samokhin, Maxim Zelikman, Vadim Tkachenko, Viktoria Bets, Elena Arzhanova, Ekaterina Litvinova

**Affiliations:** 1 Faculty of Physical Engineering, Novosibirsk State Technical University, 630073 Novosibirsk, Russia; blinovaelena-85@yandex.ru (E.B.); akorel@gmail.com (A.K.); vish22@yandex.ru (V.B.); e.arzhanova@g.nsu.ru (E.A.); dimkit@mail.ru (E.L.); 2 Institute of Organic Synthesis n.a. I. Ya. Postovsky UB RAS, 620137 Ekaterinburg, Russia; kottazem@mail.ru (E.Z.); pestov@ios.uran.ru (A.P.); 3 Institute of Solid State Chemistry and Mechanochemistry SB RAS, 630090 Novosibirsk, Russia; zelikman_mv@mail.ru; 4 Institute of Nuclear Physics SB RAS, 630090 Novosibirsk, Russia; vtkachen@mail.ru

**Keywords:** cryogel, hydrogel, carboxyethylchitosan, crosslink ratio, swelling, degradation, cytotoxicity

## Abstract

**Background:** The use of chitosan-based gels is still limited due to their restricted solubility in acid solutions, where the molecules have a positive charge. The functionalization of chitosan makes it possible to significantly expand the possibilities of using both the polymer itself and hydrogels based on its derivatives. **Objective:** To evaluate the effect of the conditions for the production of cryo- and hydrogels based on carboxyethylchitosan (CEC) crosslinked with glutaraldehyde on gel swelling and its resistance to degradation depending on pH and cytotoxic effects and to test the hypothesis that the amount of crosslinking agent during synthesis may affect the cytotoxicity of the gel. **Methods:** Gels’ swelling values and degradation resistance were determined using the gravimetric method. The cytotoxic effect was evaluated during the co-cultivation of gels in the presence of human fibroblasts using light optical microscopy and flow cytometry. **Results:** All CEC-based cryogels had a higher equilibrium swelling value and degradation time than the CEC hydrogel in the pH range from 4.6 to 8.0. This demonstrates the superiority of cryogels relative to CEC-based hydrogels in terms of swelling potential and degradation resistance, while an increase in the number of crosslinks with glutaraldehyde contributes to longer swelling of the cryogel. The positive control (intact fibroblasts) and all gel samples were statistically identical in the number of viable cells. On the third day, the viability of the fibroblast cells was consistently high (above 95%) and did not differ between all tested CEC-based gels. And in general, the cell morphology analysis results corresponded with the results obtained in the flow cytometry-based cytotoxicity test. We also did not find proof in our experiment to support our hypothesis that the amount of crosslinking agent during synthesis may affect the cytotoxicity of the material.

## 1. Introduction

Currently, requests from various industries, including the agro-industrial complex and medicine, have led to the production of a large number of gels from synthetic and natural materials. The emergence of new samples of gels as universal carriers opens up great prospects for their application to a wide range of tasks, ranging from agriculture to biotechnology and medicine [1,2,3,4,5].

Moreover, when creating various implants, carriers for drug delivery to various treatment sites in living organisms, and scaffolds for tissue engineering, preference is more often given to hydrogels from raw materials of natural origin [2,6,7,8]. One of these natural polymers is chitosan, known for its biocompatibility, lack of toxicity, sorption properties, and biodegradation ability due to the presence of amino, hydroxyl, and ester functional groups [2,9].

The degree of deacetylation and molecular weight of chitosan determine its solubility and the viscosity of aqueous solutions. This is reflected in the conditions for the formation of hydrogels. However, the use of chitosan for gel preparation is limited by the solubility of chitosan in acids and its positive charge, so researchers have carried out various modifications of chitosan to change the properties of the gels in the desired direction. Chemical modifications are mainly carried out to facilitate the solubility of chitosan in water by introducing hydrophilic groups and obtaining pH-sensitive materials [10]. Acylation, carboxylation, acrylation, benzoylation, alkylation, and quaternization of chitosan are widely used [10,11], as well as the thiolation, phosphorylation, and modification of chitosan through the use of crosslinking agents [11].

In addition to chemical modifications, physical approaches also allow for changes in the properties of chitosan gels, one of which is freezing followed by lyophilization, which aims to increase gel porosity and probably achieves very high swelling values. In this way, cryogels are obtained, which are characterized as interconnected macroporous structures, thanks to which liquid can penetrate into the gel and freely exit [12,13]. The macroporous morphology of cryogels is obtained as a result of the formation of ice crystals during cryotope gelation [14,15,16]. The diameter of the pores formed and the hydrodynamic characteristics are directly related to the temperature at which cryotope gelation is performed. The temperature primarily determines the growth time of ice crystals [17] and the rate of crosslink formation of macromolecules. Thus, by varying these parameters, cryogels based on chitosan crosslinked with glutaraldehyde can be obtained with a successfully controlled pore diameter of 60 to 240 microns [17]. In addition, the pore structure of cryogels can vary depending on the concentration of the monomer/polymer and the crosslinking agent [18,19,20], inert additives [21] and even the freeze-drying pressure [22].

Despite the fact that the macroporous structure alters the gel’s swelling ability and allows the cryogel to quickly regain its shape due to water absorption [15], it is also responsible for its relatively weak mechanical strength [13,23]. A gel’s properties can be improved by crosslinking the chitosan biopolymer with various crosslinking agents. Glutaraldehyde is often used as a crosslinking agent in the formation of cryogel from chitosan since it has high reactivity in acidic media [24,25]. It has been shown that as a result of the crosslinking process of chitosan with glutaraldehyde, the permeability, wetting, mechanical properties, and chemical stability of the gel are improved [26]. Diglycidyl esters have been proposed as an alternative to glutaraldehyde; however, the effectiveness of the interaction between chitosan and diglycidyl esters of glycols significantly depends on the nature of the acid used to dissolve the chitosan and the pH [27].

In this work, we primarily aimed to determine the effect of the conditions for the production of cryo- and hydrogels based on carboxyethylchitosan crosslinked with glutaraldehyde on gel swelling and resistance to degradation depending on pH. And the hypothesis that the amount of crosslinking agent during the synthesis process may affect the cytotoxicity of the material was the secondary aim of the study.

## 2. Results and Discussion

### 2.1. Stability and Swelling Properties of Sample 2419 Gel and Its Modifications—Samples 2711, 2712, and 2715 at Different pH Values at Room Temperature

Chitosan-based gels are characterized by the ability to swell in aqueous solutions, increase in size, and eventually degrade, but at different pH values, their ability to swell and degrade may vary.

**Figure 1:**
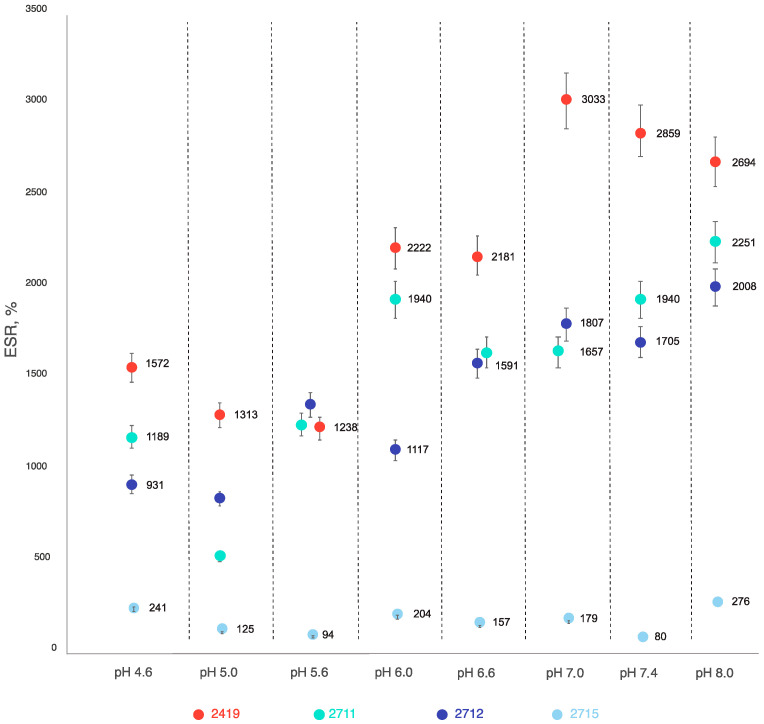
Tested CEC gels’ equilibrium swelling ratios at different pH values at 25 °C.

Despite some fluctuations in the measured ESR values caused by difficulties in maintaining standardized drying of the samples before measurement, almost all tested CEC hydrogels demonstrated that an equilibrium swelling state occurs mainly during the first 24 h. We also noted a linear association between pH and swelling ratio increase. But in the case of the CEC hydrogel (sample 2715), the degree of swelling was independent of pH (Figure 1).

The observed association between the degree of swelling and pH is due to the polycarboxylic nature of the CEC. As the pH value increases, the degree of dissociation of carboxylic groups increases, which leads to an increase in their hydrophilicity and degree of hydration. This behavior is typical for most polyelectrolytes with carboxyl groups. In addition, the data obtained confirm a comparable actual degree of crosslinking for the cryogel samples, which does not depend on the amount of crosslinking reagent under the conditions studied, which allows for the use of a minimum amount of it in the future. At the same time, the CEC crosslinking at 20 °C ensures the formation of a denser mesh structure, which affects both the lower degree of swelling of hydrogel sample 2715 and the absence of its dependence on pH (Figure 1).

Further observations of the swelling properties of the samples showed that on the second day, the equilibrium swelling of cryogel sample 2419 obtained at pH = 4.6, that of hydrogel sample 2715 at pH = 6.0 and 6.6, and that of cryogel sample 2712 at pH = 6.6 and 7.0, respectively. At pH = 8.0, the time to achieve equilibrium swelling increased for all variants of the cryogels studied: 7 days for the cryogel crosslinked with glutaraldehyde at a CEC: aldehyde molar ratio of 20:1 (sample 2711), 4 days—10:1 (sample 2419), and 3 days—5:1 (sample 2712). Thus, an increase in the amount of glutaraldehyde contributes to longer swelling of the CEC-based cryogel under alkaline conditions (pH = 8.0). But according to the elemental analysis and FT IR spectroscopy results, it was not possible to establish a significant difference in the degree of crosslinking, and the kinetic properties of swelling are more sensitive to the crosslinking density.

As a result, the data obtained unambiguously demonstrate the advantages of cryogels obtained by freezing an aqueous reaction mass (−19 °C), since all of the studied CEC-based cryogels had a higher equilibrium swelling value than the hydrogel in which crosslinking was carried out at +20 °C in the pH range of 4.6 to 8.0.

This demonstrates the superiority of cryogels compared to hydrogels in terms of swelling potential. The main reasons for this are the lower actual degree of crosslinking and, as a result, the lower mesh density of covalent intermolecular bonds and the high macroporosity of cryogels compared to hydrogels, which accelerates diffusion processes during solvent sorption by the gel.

### 2.2. Evaluation of the Gels’ Resistance to Degradation Properties

The obtained gels were synthesized with the aim of achieving a high swelling ratio; thus, it is not surprising that the gels were able to increase their mass to thousands of percent, and some gel samples achieved maximum swelling values within several days from the beginning of the experiment and then started to degrade (i.e., lose their mass).

**Figure 2:**
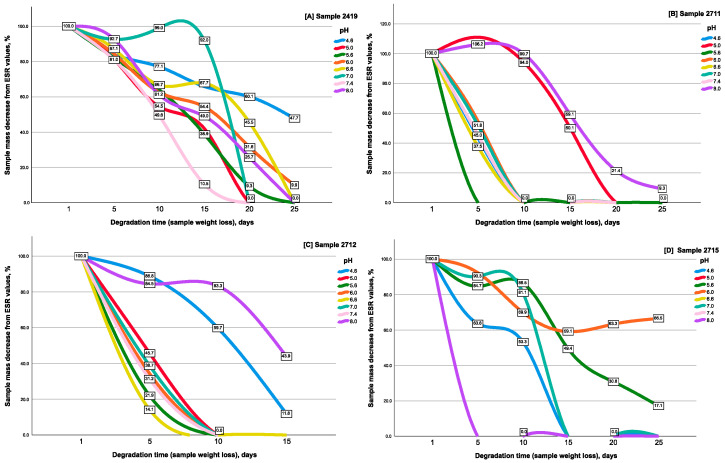
Degradation kinetics of the CEC cryogel and hydrogel samples, tested at room temperature and different pH values (all data presented as sample mass decrease from ESR values).

Degradation of the CEC hydrogel (sample 2715) mainly occurred on day 3 (for pH values = 5.0, 6.6, 7.4, and 8.0), and at pH = 4.6 and 7.0—on day 15. This hydrogel sample was most stable at pH = 5.6 and 6.0, and its degradation time was 24 and 30 days, respectively (Figure 2D).

The CEC cryogels showed more expressed degradation resistance—depending on the amount of crosslinking agent and the pH value, the minimum degradation time ranged from 7 to 15 days. Thus, for the cryogel crosslinked with glutaraldehyde at a CEC: aldehyde molar ratio of 10:1 (sample 2419) (Figure 2A), the degradation time at pH = 7.4 was 15 days, and at pH = 5.0, it was 5.6–17 days. This cryogel was more resistant to degradation at pH = 6.0 and 6.6. Its degradation occurred on the 21st–23rd day of incubation. The maximum resistance to degradation of the cryogel crosslinked at a ratio of 10:1 was observed under acidic conditions (pH = 4.6) for 40 days (Figure 2A), which is the longest degradation period of all tested gel samples in the studied pH range.

Degradation of the CEC cryogel crosslinked with glutaraldehyde at a CEC: aldehyde molar ratio of 20:1 (sample 2711) (Figure 2B) for most pH values already occurred on day 8. The exception was the degradation time at pH = 5.0 and 8.0, which occurred on days 16 and 25, respectively.

Degradation of the cryogel crosslinked with glutaraldehyde at a ratio of 5:1 (sample 2712) (Figure 2C) occurred within 1–2 weeks: on day 7 in the pH range = 5.6–6.6 and on day 8 under neutral conditions of pH = 7.0 and 7.4. At pH = 4.6, degradation was recorded on day 15 and on day 16—at pH = 8.0. Interestingly, the degradation time of cryogels under alkaline conditions (pH = 8.0) increased with the increase in the amount of crosslinking agent (16 days at a ratio of 5:1, whereas at a ratio of 10:1 and 20:1, it was 21 and 25 days, respectively). For the degradation time under acidic conditions (pH = 4.6), no such pattern was observed; however, the maximum degradation resistance of the cryogel sample crosslinked with glutaraldehyde at a CEC: aldehyde molar ratio of 10:1 (sample 2419) was revealed under these pH conditions, which significantly differed from the stability of the other CEC modifications.

**Figure 3:**
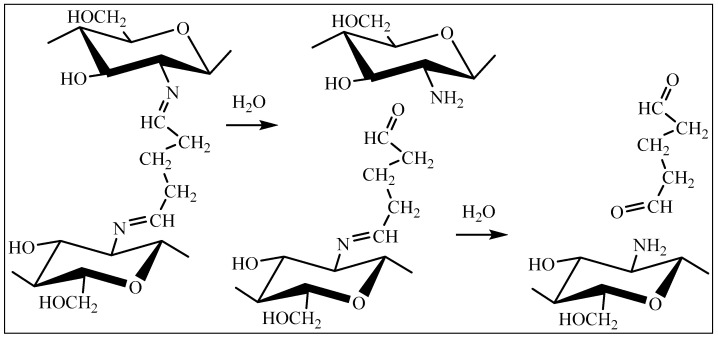
Scheme of hydrolysis of the amino group of the carboxyethylchitosan hydrogel crosslinked with glutaraldehyde.

The fundamentally different observed kinetic dependences of hydrogel degradation on pH also seem to demonstrate the capabilities of this method in assessing the features of the supramolecular structure of hydrogels, depending on the conditions of their formation. The main reaction that ensures the degradation of hydrogels is the hydrolysis of amino groups (Figure 3) formed during the crosslinking of CEC.

Thus, the CEC cryogel crosslinked with glutaraldehyde at a CEC: aldehyde molar ratio of 10:1 has more uniform time intervals of swelling and degradation, which allows for standardized loading of the cryogel with drugs or other agents. Its special resistance in acidic conditions can find various industrial applications, including when used as a carrier for bacterial and humic preparations for subsequent application to acidic soils.

### 2.3. Gel Sample Cytotoxicity Evaluation on Human Fibroblast Culture In Vitro

Since we used glutaraldehyde as a crosslinking agent, which may be a toxic substance according to the available literature [28,29], we performed an in vitro evaluation of the cytotoxic properties of glutaraldehyde in various concentrations for both CEC hydrogels and cryogels. We tested samples of CEC gels for cytotoxicity against human fibroblast culture for 3 days.

#### 2.3.1. Cell Line Morphology Results

**Figure 4:**
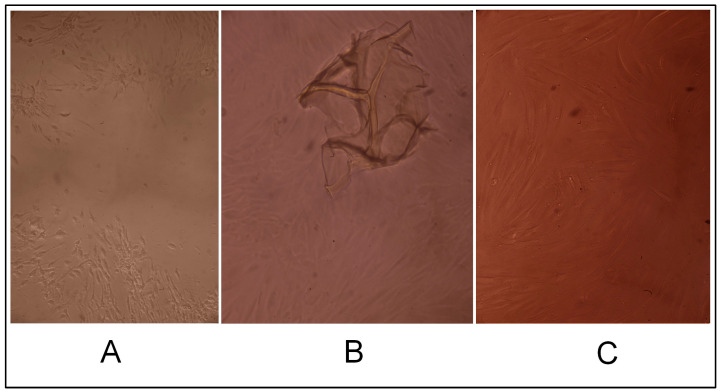
Cytotoxic effect of the cryogel CEC samples crosslinked with glutaraldehyde at a CEC: aldehyde molar ratio of 10:1 (sample 2419) per cell: (**A**)—1 day, (**B**)—2 days, and (**C**)—3 days; phase contrast, ×20.

The hydrogel CEC crosslinked with glutaraldehyde at a CEC: aldehyde molar ratio of 10:1, according to the results of cell morphology analysis, had a slight cytotoxic effect: a small cell-free field was present in the center of the cell monolayer (Figure 4B,C).

**Figure 5:**
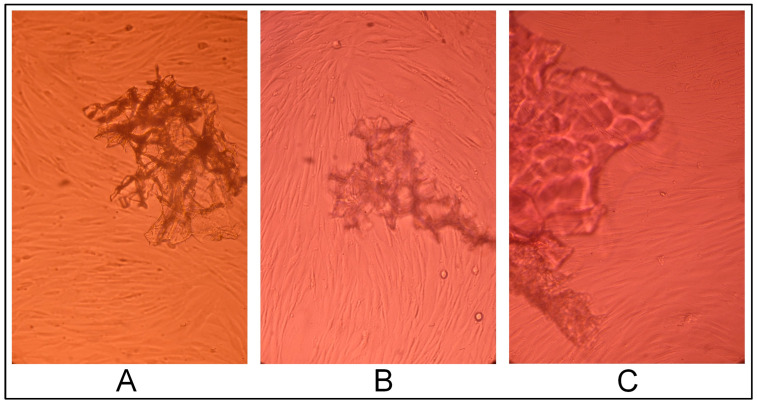
Cytotoxic effect of the cryogel CEC samples crosslinked with glutaraldehyde at a CEC: aldehyde molar ratio of 20:1 (sample 2711) on cells: (**A**)—1 day, (**B**)—2 days, and (**C**)—3 days; phase contrast, ×20.

**Figure 6:**
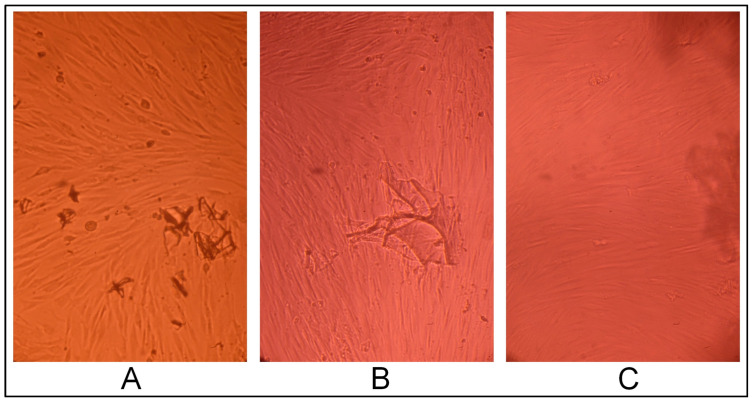
Cytotoxic effect of the cryogel CEC samples crosslinked with glutaraldehyde at a CEC: aldehyde molar ratio of 5:1 (sample 2712) on cells: (**A**)—1 day, (**B**)—2 days, and (**C**)—3 days; phase contrast, ×20.

**Figure 7:**
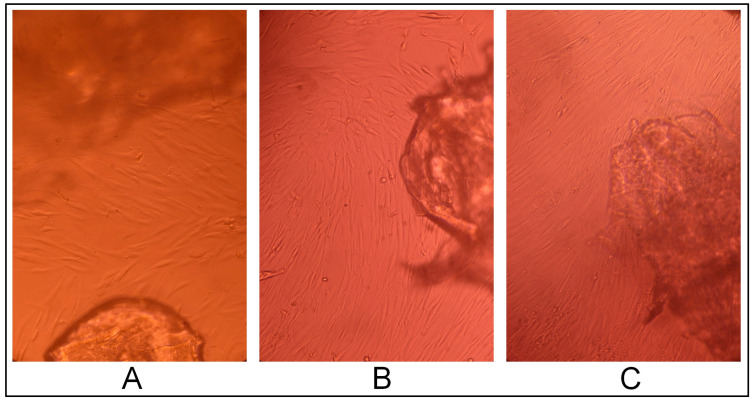
Cytotoxic effect of the CEC hydrogel samples crosslinked with glutaraldehyde at a CEC: aldehyde molar ratio of 10:1 (sample 2715) on cells: (**A**)—1 day, (**B**)—2 days, and (**C**)—3 days; phase contrast, ×20.

**Figure 8:**
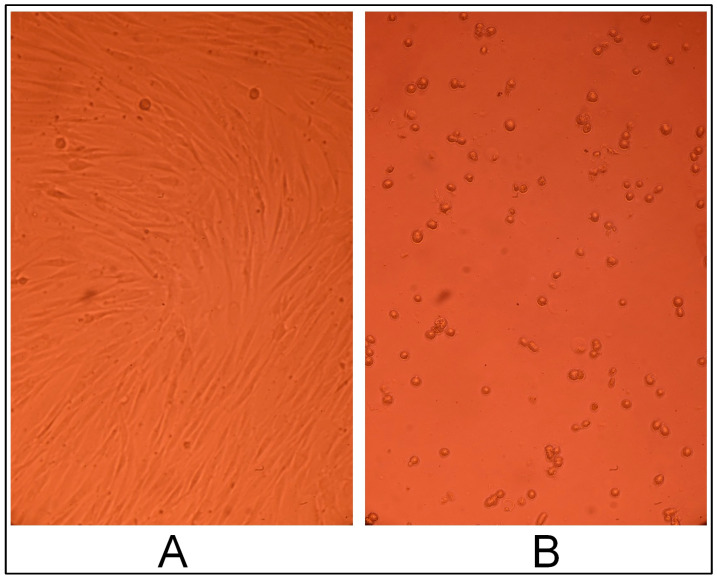
Cytotoxic effect in the controls. (**A**)—positive control and (**B**)—negative control.

CEC cryogels synthesized using glutaraldehyde as the crosslinking agent in ratios 5:1, 10:1, and 20:1 (sample 2712, sample 2419, and sample 2711, respectively) did not have a cytotoxic effect on the fibroblast culture; the cells had a typical morphology and proliferated to form a confluent monolayer for three days (Figure 5, Figure 6 and Figure 7), and were morphologically the same as the morphology of the cells in the positive control (intact fibroblasts) (Figure 8A).

On the contrary, cells in the negative control (treated with DMSO) acquired a spherical shape (i.e., rounded cells); some were in the stage of detaching from the surface of the culture plate well, and cell-free fields were observed (Figure 8B).

#### 2.3.2. Cell Line Flow Cytometry-Based Cytotoxicity Results

In general, the results of the cell morphology analysis corresponded to the results obtained in the flow cytometry-based cytotoxicity assay using Calcein-AM and 7-AAD dyes. The number of viable cells was determined as the percentage of Calcein-AM and 7-AAD cells stained with the used dyes in the fibroblast gate. Intact fibroblasts were used as a positive control, and fibroblasts cultured in the presence of 10% DMSO were used as a negative control.

**Figure 9:**
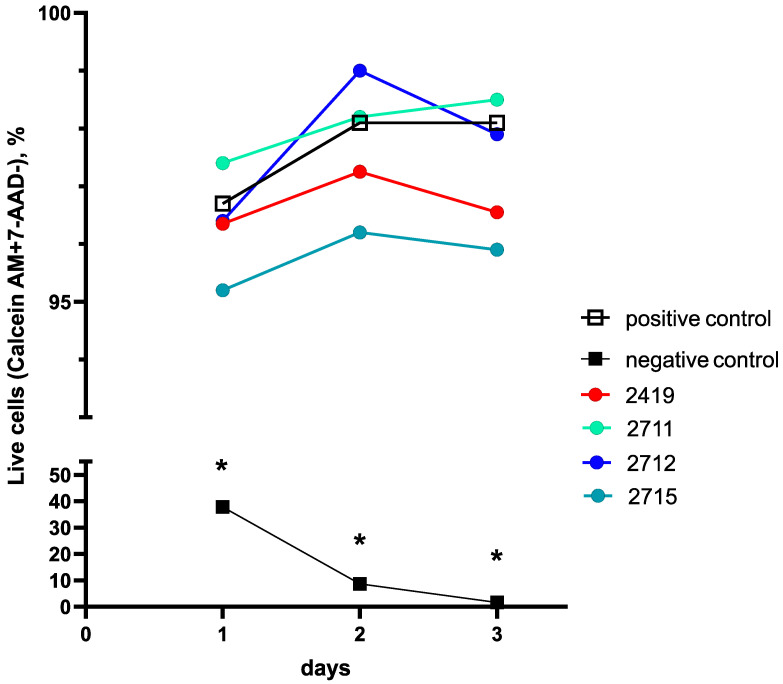
Human fibroblast cell viability evaluation using the flow cytometry-based cytotoxicity assay with Calcein-AM and 7-AAD fluorescent dyes staining for the CEC gel sample 2419 and its modifications (sample 2711, sample 2712, and sample 2715) on human fibroblast cells in vitro. Note: *—significant differences relative to the positive control, *p* < 0.01 (corrected alpha = 0.01). All data are presented as medians. The experiment was performed in triplicate. Positive control—the intact fibroblasts; negative control—fibroblasts cultured in the presence of 10% DMSO.

The proportion of viable fibroblasts in either the positive control or in the cryogel and the hydrogel CEC samples was about 95–99% (Figure 9).

All tested cryogel samples had no cytotoxic effect on fibroblasts for 3 days, and the percentage of live cells did not differ from those in the positive control except for hydrogel sample 2715, but this difference was practically negligible. For the last gel on the third day, we observed a slight “statistical” cytotoxic effect (98.1% [97.9; 98.7] in the positive control versus 95.9% [94.9; 96.0] for sample 2715 (*p* = 0.017). Taking into account that these results were obtained during multiple comparisons with Bonferroni correction (corrected alpha = 0.016), the statistical significance was absent.

On the contrary, for the negative control, significant differences in the number of viable fibroblasts compared with the positive control were noted on all days of the study (Figure 9) and within the negative control itself.

Indeed, the percentage of live fibroblast cells in the negative control between day 1 and day 3 diminished dramatically by almost 23 times—from the initial 37.8% [21.30; 48.00] to near zero 1.70% [1.10; 3.00] (Friedman test, *p* = 0.00013), i.e., cell culture in the negative control died during the experiment.

At the same time, differences between negative and positive controls during the whole experiment were always statistically significant—from 37.8 [21.30; 48.00] and 96.7 [95.60; 98.80] (*p* = 0.001, corrected alpha = 0.01) on day 1 for the negative and positive controls, respectively, to 1.70% [1.10; 3.00] and 98.1 [97.20; 98.70] (*p* = 0.001, corrected alpha = 0.01) on day 3, respectively (Figure 9).

It should be noted that the difference between the positive and negative controls during the experiment was always measured with the value of at least *p* = 0.002 (corrected alpha = 0.01), thus always being significant, which was also confirmed by the results of the cell morphology analysis described in the Section 2.3.1.

Nevertheless, we did not find any significant differences in the number of live cells between the positive control and all tested gels during the whole experiment (corrected alpha > 0.01); thus, the positive control and all gel samples were statistically identical in the number of viable cells.

For example, CEC cryogel sample 2712 (with a CEC: glutaraldehyde molar ratio of 5:1) demonstrated an increase (Friedman test, *p* = 0.028; corrected alpha = 0.016) in the proportion of viable cells in culture on day 2—from 96.4% [96.1; 96.8] to 99.0% [98.30; 99.20] and stabilized at 97.9% [96.40; 98.50] on day 3, with this being similar to the positive control.

A comparison between the different gel samples during the experiment showed some statistically significant results, but these differences are of statistical importance only, and all gels showed very good cell viability (Figure 9).

In particular, on day 2, the viability of the fibroblasts in the presence of sample 2712 in comparison with sample 2419 was high enough in both cases (*p* = 0.024; corrected alpha = 0.01). A detected difference was only found in the trend curve fluctuation (Figure 13), rather than being practical of interest, since the number of viable cells was consistently high (more than 95%) in both cases and the exact *p*-level lies above the corrected alpha value.

Previously published studies have shown that glutaraldehyde, widely used as a crosslinking agent in the formation of chitosan scaffolds, contributes to the toxicity of a biomaterial when its content exceeds, according to various data, 10 mol% [28] or more than 8% [29]. In some studies [30], it has also been shown that the cytotoxic effect of glutaraldehyde when used for crosslinking CEC has a molar dependence, with an increase in toxicity proportional to an increase in crosslinking density.

In our study, on day 3 in the cell culture, a slight cytotoxic effect was noted during cell morphology analysis for the hydrogel form of CEC crosslinked with glutaraldehyde at a CEC: aldehyde molar ratio of 10:1 (sample 2715) only.

Nevertheless, on the third day, the viability of fibroblast cells was consistently high (above 95%) and did not differ between all tested CEC-based gels. Thus, the obtained results meant that we were unable to confirm the hypothesis that the amount of crosslinking agent during synthesis may affect the cytotoxicity of the material.

## 3. Conclusions

It was shown that all CEC-based cryogels had a higher equilibrium swelling ratio than the hydrogel in the pH range of 4.6 to 8.0. This demonstrates the superiority of cryogels compared to hydrogels in terms of swelling potential, while an increase in the amount of crosslinking with glutaraldehyde contributes to longer swelling of the cryogel.

For most pH values, degradation of the CEC hydrogel (sample 2715) occurred within three days, whereas CEC cryogels showed more pronounced resistance to degradation—depending on the crosslinking agent amount added during synthesis and the pH value of the buffer medium, the minimum degradation time for cryogels varied from 7 to 15 days.

Evaluation of the cytotoxic effect of gels in human fibroblast cell culture using cell morphology analysis showed that on day 3 of cultivation, only a slight cytotoxic effect was noted for the hydrogel form of CEC crosslinked with glutaraldehyde at a CEC: aldehyde molar ratio of 10:1 (sample 2715). Nevertheless, we did not find any significant differences in the number of live cells between the positive control and all tested gels during the whole experiment (corrected alpha > 0.01); thus, the positive control and all gel samples were statistically identical in the number of viable cells. On the third day, the viability of fibroblast cells was consistently high (above 95%) and did not differ between all tested CEC-based gels. And in general, the cell morphology analysis results corresponded with the results obtained in the flow cytometry-based cytotoxicity test.

We also did not find any proof in our experiment to support our hypothesis that the amount of crosslinking agent during synthesis may affect the cytotoxicity of the material.

## 4. Materials and Methods

### 4.1. Bioresorbable Gel Carrier

**Figure 10:**
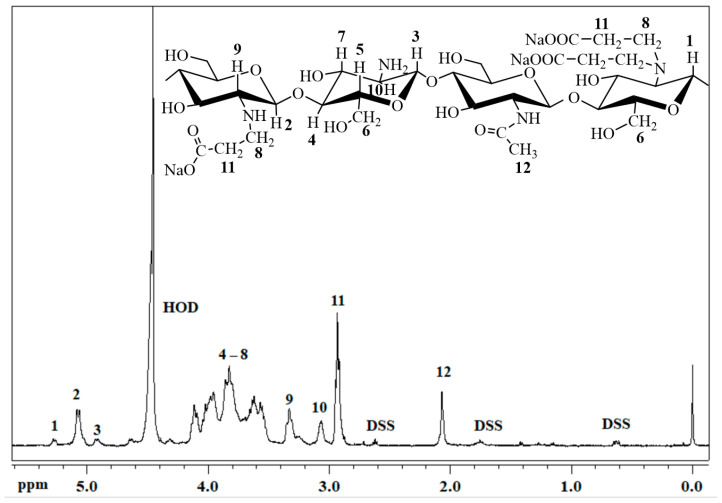
NMR ^1^H spectrum of CEC (D_2_O/DCl).

**Figure 11:**
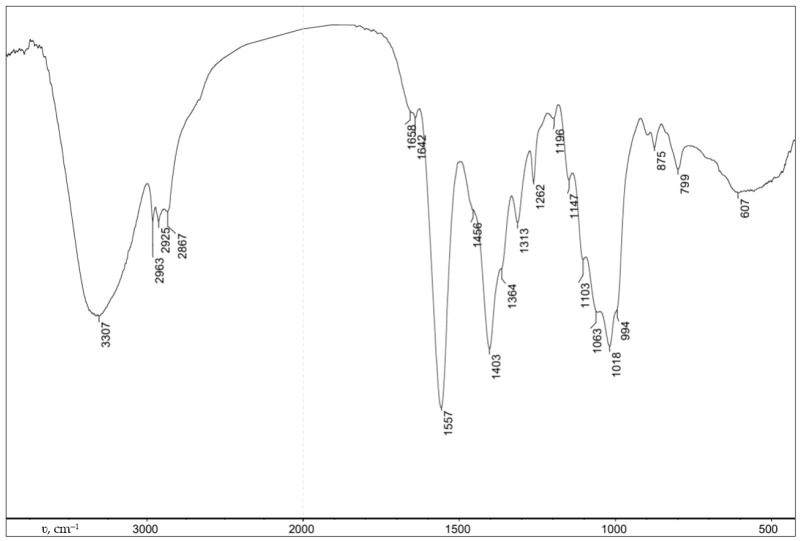
FTIR spectrum of CEC.

Carboxyethyl chitosan (CEC) was obtained using a previously developed method [31], with the usage of chitosan with 86% deacetylation (Orison Chemicals Limited, Tianjin, China) and glutaraldehyde 50% water solution (Ningxia Jinghong Chemical Co., Ltd., Ningxia, China). The degree of substitution of amino groups was 1. The composition and structure of the obtained CEC were confirmed using data from elemental analysis. The elemental analysis was performed using the automatic analyzer Perkin Elmer CHN PE 2400 (Perkin Elmer, Waltham, MA, USA), IR spectroscopy spectra were recorded using a Spectrum Two spectrometer (Perkin Elmer, Waltham, MA, USA), and spectra using NMR 1H spectroscopy were recorded on a Bruker Avance DRX-400 spectrometer (Bruker Biospin, Rheinstetten, Germany) (Figure 10 and Figure 11).

**Figure 12:**
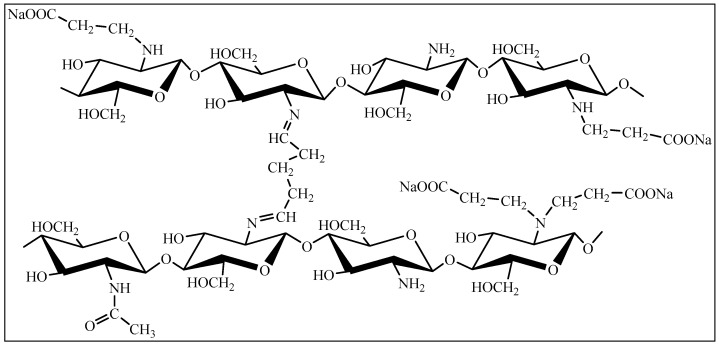
Chemical structure fragment of CEC crosslinked with glutaraldehyde.

The hydrogel materials are samples of swollen CEC, crosslinked with glutaraldehyde at different CEC: aldehyde molar ratios (Figure 12).

To obtain gels, a 3% polymer solution in acetic acid at pH = 6 and glutaraldehyde were used at a CEC: aldehyde molar ratio of 10:1. Crosslinking was performed at 20 °C (sample 2715) and −19 °C (sample 2419). To assess the effect of the amount of crosslinking agent, crosslinking was performed at a CEC: aldehyde molar ratio of 5:1 (sample 2712) and 20:1 (sample 2711), both at −19 °C.

At least four gel samples were synthesized, with each sample including three specimens:

1. Sample 2419—CEC cryogel crosslinked with glutaraldehyde (CEC: aldehyde molar ratio of 10:1).
2. Sample 2715—CEC hydrogel crosslinked with glutaraldehyde (CEC: aldehyde molar ratio of 10:1).
3. Sample 2712—CEC cryogel crosslinked with glutaraldehyde (CEC: aldehyde molar ratio of 5:1).
4. Sample 2711—CEC cryogel crosslinked with glutaraldehyde (CEC: aldehyde molar ratio of 20:1).

**Figure 13:**
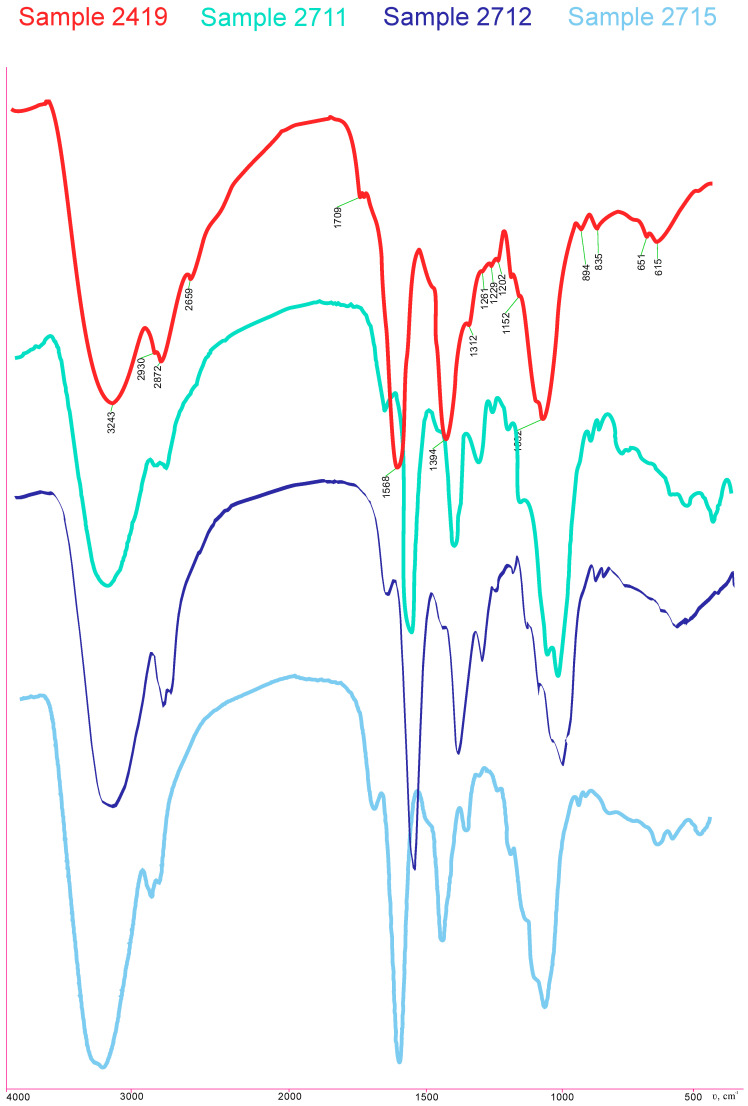
FTIR spectra of samples 2419, 2711, 2712, and 2715.

The composition and structure of the obtained CEC cryogel and CEC hydrogels were confirmed using data from elemental analysis, IR spectroscopy, and NMR ^1^H spectroscopy (Figure 13).

During the formation of the hydrogel material, the CEC molecules crosslink due to the interaction of the primary amino groups of the polymer with the aldehyde groups of the crosslinking agent (Figure 12). Regarding the elemental analysis results, the degree of crosslinking functionalization was no more than 3% molar, which demonstrates a negligible effect of the amount of crosslinking agent on actual crosslinking for the studied polymer: aldehyde ratios.

Indeed, the IR spectroscopy results (Figure 13) show intense absorption bands at 3277 (O−H and N−H), 2873 (C−H), 1564 and 1397 (COO−), and 1059 and 1023 (C−O, C−C) cm^−1^ with the presence of an absorption band at 1642 cm^−1^ corresponding to the imine group (C=N), which was formed as a result of the interaction between the primary amino groups of CEC and glutaraldehyde (Figure 12). The ratio of absorption band intensities also demonstrates comparable values to the actual crosslinking of macromolecules, regardless of the polymer: crosslinking agent ratio.

Before the experiment, the gels were sterilized according to the previously selected method, electron beam sterilization with an absorbed dose of 25 kGy [32], and the experiment was carried out using sterile samples only.

### 4.2. Swelling Analysis and Degradation Evaluation of CEC Gels under Various pHs

The swelling properties were measured using the gravimetric method, with the pH in the range of 4.6–8.0 (with 0.4–0.5 increments between pH values) and constant temperature mode at room temperature.

Gel samples pre-dried at 25 °C (about 3 mm^3^) were always weighed before swelling measurement and then immersed in 10 mL of phosphate-buffered saline (PBS) in 10 cm Petri dishes. For 40 days or until full degradation, all samples were weighed at least once a day and then returned to the PBS. The corresponding pH buffer solutions were prepared based on the method of Mcilvaine T.C. [33].

The swelling ratio (SR) and equilibrium swelling ratio (ESR) were calculated using the following formulas:

SR (%) = (m_t_ − m_0_)/m_0_ × 100% (1)

ESR (%) = (m_∞_ − m_0_)/m_0_ × 100% (2)

where m_0_ is the mass of dry gel, m_t_ is the mass of swollen gel at time t, and m_∞_ is the mass of the gel at equilibrium swelling.

### 4.3. Cytotoxicity Evaluation

The effect of the gel on the viability of human fibroblasts was evaluated after 24, 48, and 72 h of incubation with cells (confluent monolayer) in 12-well plates (TPP, Trasadingen, Switzerland). The cells were seeded in plates (at least 6 × 10^4^ cells per cm^2^) using the medium, which consisted of DMEM (Gibco, Carlsbad, CA, USA) with 10% fetal serum (StemCell Technologies, Vancouver, BC, Canada), 2 mM of L-glutamine (StemCell Technologies, Canada) and combined antibiotics of 100 IU/mL of penicillin and 100 μg/mL of streptomycin (Biolot, St. Petersburg, Russia). The plates were incubated in a CO_2_ incubator at +37 °C in air with 5% CO_2_. After reaching the confluent monolayer, sterile gel samples (approximately 3 mm^3^ in size) were placed on top of the cell monolayer (study group, *n* = 4). Cultivation of samples with cells was carried out in three replicates.

Fibroblasts cultured without gel samples and any other medium components were used as a positive control (*n* = 7). Fibroblasts cultured in addition to 10% DMSO (Biolot, St. Petersburg, Russia) were used as negative controls (*n* = 4). At the end of the incubation period, the samples were removed, and the cells were washed with Dulbecco’s phosphate-buffered saline solution (DPBS) (Gibco, Carlsbad, CA, USA). Afterward, the cells were detached with a 1:1 trypsin: Versen solution (Biolot, St. Petersburg, Russia) and centrifuged for 10 min at 1100 rpm. The pellet was resuspended in the complete medium. Next, the cells were centrifuged for 5 min at 1100 rpm and then washed with 1 mL of PBS. Afterward, the cell pellet was added to 500 µL of DPBS (Gibco, Carlsbad, CA, USA) containing 100 nM of the fluorescent dye Calcein AM Viability Dye (eBioscience Invitrogen, San Diego, CA, USA) for visualization of living cells, followed by incubation for 15 min in the dark at room temperature. Then, 5 µL of fluorescent dye 7-AAD Viability Staining Solution (eBioscience Invitrogen, San Diego, CA, USA) was added to the solution to visualize dead cells, and they were stained for 5 min in the dark at room temperature. For the detection and quantitative analysis of living and dead cells, samples stained with the above dyes were analyzed on a FACS Canto II flow cytometer (Becton Dickinson, Franklin Lakes, NJ, USA) using FACS Diva 6.0 software (Becton Dickinson, Franklin Lakes, NJ, USA).

The cell culture was also examined using inverted light microscopy analysis of the culture plates to visualize cell growth conditions.

### 4.4. Statistics

Statistical analysis was performed using GraphPad Prism software (version 9.0) and IBM SPSS software (version 25.0). For discrete variables, a comparison was performed using non-parametric tests: the Friedman test and the Mann–Whitney test with exact *p*-value calculation and Bonferroni correction for multiple comparisons. All discrete data are presented as medians and quartiles. Significance levels are indicated either as absolute values or as *p* < 0.01.

## Data Availability

The raw data supporting the conclusions of this article will be made available by the authors on request.
